# Evaluation of DNA damage in COPD patients and its correlation with polymorphisms in repair genes

**DOI:** 10.1186/1471-2350-14-93

**Published:** 2013-09-20

**Authors:** Andréa Lúcia Gonçalves da Silva, Helen Tais da Rosa, Thaís Evelyn Karnopp, Clara Forrer Charlier, Joel Henrique Ellwanger, Dinara Jaqueline Moura, Lia Gonçalves Possuelo, Andréia Rosane de Moura Valim, Temenouga Nikolova Guecheva, João Antonio Pêgas Henriques

**Affiliations:** 1 Santa Cruz Hospital and Department of Health and Physical Education, University of Santa Cruz do Sul - UNISC, Avenida Independência, 2293, Bloco 42, Bairro Universitário, Santa Cruz do Sul, RS, Brazil; 2 Graduate Program in Cell and Molecular Biology, Federal University of Rio Grande do Sul - UFRGS, Porto Alegre, RS, Brazil; 3 Scientific Initiation of University of Santa Cruz do Sul - UNISC, Santa Cruz do Sul, RS, Brazil; 4 Department of Biology and Pharmacy, University of Santa Cruz do Sul - UNISC, Santa Cruz do Sul, RS, Brazil; 5 Laboratory of Genetic Toxicology, Federal University of Health Sciences of Porto Alegre - UFCSPA, Porto Alegre, RS, Brazil; 6 Department of Biophysics, Federal University of Rio Grande do Sul - UFRGS, Porto Alegre, RS, Brazil; 7 Institute of Biotechnology, University of Caxias do Sul, Caxias do Sul, RS, Brazil

**Keywords:** COPD, DNA damage, DNA repair, Genetic polymorphisms, BMCyt

## Abstract

**Background:**

We investigated a potential link between genetic polymorphisms in genes *XRCC1* (Arg399Gln), *OGG1* (Ser326Cys), *XRCC3* (Thr241Met), and *XRCC4* (Ile401Thr) with the level of DNA damage and repair, accessed by comet and micronucleus test, in 51 COPD patients and 51 controls.

**Methods:**

Peripheral blood was used to perform the alkaline and neutral comet assay; and genetic polymorphisms by PCR/RFLP. To assess the susceptibility to exogenous DNA damage, the cells were treated with methyl methanesulphonate for 1-h or 3-h. After 3-h treatment the % residual damage was calculated assuming the value of 1-h treatment as 100%. The cytogenetic damage was evaluated by buccal micronucleus cytome assay (BMCyt).

**Results:**

COPD patients with the risk allele *XRCC1* (Arg399Gln) and *XRCC3* (Thr241Met) showed higher DNA damage by comet assay. The residual damage was higher for COPD with risk allele in the four genes. In COPD patients was showed negative correlation between BMCyt (binucleated, nuclear bud, condensed chromatin and karyorrhexic cells) with pulmonary function and some variant genotypes.

**Conclusion:**

Our results suggest a possible association between variant genotypes in *XRCC1* (Arg399Gln), *OGG1* (Ser326Cys), *XRCC3* (Thr241Met), and *XRCC4* (Ile401Thr), DNA damage and progression of COPD.

## Background

Recently genetic association studies on chronic obstructive pulmonary disease (COPD) risk have focused on identifying effects of single nucleotide polymorphisms/haplotypes in candidate genes, among which DNA repair genes are increasingly studied because of their critical role in maintaining genome integrity [1]. Assays that measure DNA repair capacity suggest that it can vary widely among individuals. Previous investigations reported lower DNA repair capacity in COPD patients [1-3]. The finding supports the hypothesis that variants in DNA repair genes could affect COPD susceptibility. In general, studies are needed to elucidate the mechanisms by which genes are involved in the development of COPD, lung cancer, or both as well as to understand of damage recognition and mechanism of DNA repair [2,4].

The DNA repair system consists of several distinct pathways, including the base excision repair (BER) pathway and double-strand break (DSB) repair process [5]. BER pathway is a DNA repair process which operates on small lesions such as oxidized or reduced bases, fragmented or non-bulky adducts, or those produced by methylating agents and still acting on bases deamination injury induced by environmental carcinogens or endogenous sources [1,2,6]. The genes encoding three key enzymes in this repair pathway are: 8-oxoguanine DNA glycosylase (*OGG1*), apurinic/apyrimidinic endonuclease (*APE1*/*APEX1*), and the X-ray repair cross complementing group 1 (*XRCC1*). OGG1 and XRCC1 play a central role in the BER pathway. OGG1 catalyzes the removal of 8-hydrodeoxy-guanine (8-OHdG), which has been considered as a key biomarker of oxidative DNA damage. The substitution of cysteine for serine at codon 326 (Ser326Cys) is associated with a significant reduction in the repair capacity [6]. Evidence has emerged to support that BER deficiency is an important contributing factor to cancer susceptibility, as shown in both animal models and human studies [1,2]. Hence, *OGG1* and *XRCC1* are suggested to exert combined effect on the development of COPD, and *XRCC1* coordinates and stimulates the OGG1 activity [6].

Double-stranded breaks are repaired by two pathways: homologous recombination (HR) and non-homologous DNA end joining (NHEJ) [7]. NHEJ is an intrinsically error-prone pathway while HR results in an free error repair [8]. HR and NHEJ pathways may have overlapping functions to maintain chromosomal integrity in eukaryotes [9]. X-ray repair cross complementing-3 (XRCC3) is involved in the homologous recombination pathway of DNA DSB-repair [10] and the role *XRCC3* gene have been most extensively studied due to their influences in the individual sensitivity to radiation exposure and induction of DNA damage. The X-ray repair cross complementing-4 (*XRCC4*) gene functions in the repair of DNA double-strand is also important [11] and the *XRCC4* inactivation is related to programmed cell death or apoptosis [5].

The cellular processes of DNA damage induction and repair are fundamental for the maintenance of genome integrity, and the modulation of these processes can dramatically increase individual susceptibility to cancer [12,13]. Comet assay has been used in various studies to investigate the DNA damage in connection with various diseases because it is a rapid, simple, and sensitive technique for measuring DNA breaks and repair in single cells [14-16]. The cytogenetic damage evaluated by buccal micronucleus cytome assay (BMCyt) is a sensitive biomarker that is widely accepted for chromosome damage evaluation [17]. The buccal epithelial cells are the first barrier for the inhalation or ingestion route that can metabolize proximate carcinogens to reactive products. About 90% of the human cancers originate from epithelial cells. Therefore, oral epithelial cells represent a preferred target site for early genotoxic events induced by carcinogenic agents entering the body.

We aimed to investigate a potential effect of genetic polymorphisms in genes *XRCC1* (Arg399Gln), *OGG1* (Ser326Cys), *XRCC3* (Thr241Met), and *XRCC4* (Ile401Thr) on the level of DNA damage and repair in COPD patients and controls. Accordingly, we intend to understand the role of genetic polymorphisms in the modulation of DNA damage and progression of COPD.

## Methods

Fifty-one COPD patients, treated at Santa Cruz Hospital by the Research Group for Health Rehabilitation, Santa Cruz do Sul, RS, Brazil were included in this study. COPD was diagnosed according to the Global Initiative for Chronic Obstructive Lung Disease guidelines (GOLD) [18] using clinical history, physical examination, and presence of airflow obstruction, defined as a ratio of forced expiratory volume in one second (FEV_1_) to forced vital capacity (FVC) less than 70% of predicted value. The patients were grouped in according to COPD stage as mild, moderate, severe or very severe [19]. The COPD patients were matched by gender, age and body mass index (BMI) with 51 controls without pulmonary disease. The study protocol was approved by the Ethics Committee of the University of Santa Cruz do Sul, protocol number 2011/08. All individuals answered the personal health questionnaire and signed informed consent before the interview.

### Obtaining sample

The peripheral blood (10 mL) samples were collected early in the morning from fasted COPD patients and controls into tubes with EDTA, and used to comet assay and genetic polymorphisms identification. Buccal cell samples were collected and processed in accordance with Thomas et al. [20]. For each subject were prepared two tubes for left cheek (LC) and right cheek (RC) cells, each containing methanol. The cells were collected rotating a cytobrush 20 times in a spiral motion against the inner surface of the cheek wall. The head of cytobrush was placed into the tube respective with methanol.

### DNA damage evaluation by comet assay

The comet assay was performed under alkaline (detects single- and double-DNA strand breaks and alkali-labile sites) and neutral (detects double breaks) conditions according to the procedure of Singh et al. [21] with the modifications by Tice et al. [22]. Aliquots of 10 μl freshly collected whole blood were embedded in 90 μl of 0.75% low melting agarose, and after agarose solidified slides were placed in lyses buffer (2.5 M NaCl, 100 mM EDTA, 10 mM Tris; 10% DMSO; pH 10.0-10.5) containing freshly added 1% (v/v) Triton X-100 and 10% (v/v) dimethyl sulphoxide for maximum of 2 weeks. After treatment with lyses buffer slides and placed fresh prepared alkaline buffer solution (200 mM NaOH and 1 mM EDTA; pH> 13), for 20 min, and DNA submitted of denaturation and 15 min electrophoresis time were used (0.90 V/cm and 300 mA). For the neutral condition (3 M sodium acetate and 1 M Tris; pH=8.5), the denaturation time of 1-h and electrophoresis time of 1-h were used (0.5 V/cm and 12 mA) [23]. In both versions of the comet assay, after electrophoresis, the slides were neutralized with 0.4 M Tris (pH 7.5) and the DNA fixed and stained with silver nitrate in accordance to Nadin et al. [24]. Images of 100 randomly selected cells (50 cells from each of two replicate slides) were analyzed for each individual using a conventional microscope. Two parameters were evaluated according Heuser et al. [25]. International guidelines and recommendations for the comet assay consider that visual scoring of comets is a well-validated evaluation method [22,26]. Damage index thus ranged from 0 (completely undamaged: 100 cells × 0) to 400 (with maximum damage: 100 cells × 4).

### Comet assay to assess the susceptibility to exogenous DNA damage

For the assessment of susceptibility to exogenous DNA damage, whole blood cells were treated with methylmethane sulfonate alkylating agent (MMS; 80 μM) for 1-h or 3-h at 37°C prior to slide preparation, and proceeded up the steps of the alkaline comet assay as described above. The percentage of residual DNA damage after 3-h MMS treatment was calculated using the value of 1-h MMS treatment for each subject as 100%.

### Buccal micronucleus cytome assay (BMCyt)

The cell suspensions were stored at 4°C, until processing. Afterwards, the cells were centrifuged and the supernatant was aspirated and added if more buccal cell fixation buffer. To further cellular disaggregation 5% of DMSO was added to each of cell suspension. The fixed cells were hydrolyzed in HCl and stained according Feulgen method [20]. The scoring criteria for the distinct cell types and nuclear anomalies in the BMCyt assay were intended for classifying buccal cells into categories that distinguish between ‘normal’ cells (Basal cell) and cells that are considered ‘abnormal’ on the basis of cytological and nuclear features, indicative of DNA damage (Micronucleated-MN; Nuclear Bud- BUD), cytokinesis failure (Binucleated-BI) or cell death (Condensed chromatin-CC; Karyorrhectic-KR; Pyknotic-PY; Karyolytic- KL). Two thousand cells per sample were scored to determine the frequency of these cell types [20].

### DNA extraction and genotyping

Genomic DNA was isolated from whole blood by the salting out method [27]. Four polymorphic markers were investigated by genotyping using the polymerase chain reaction (PCR) – restriction fragment length polymorph-ism (RFLP) method. In the Table 1 were show the primers pairs used, annealing temperature, fragment size and restriction enzyme of the genes studied [28-31]. All digestion were conducted with a total volume of 15 μL for 18 h at 37°C and subsequently analyzed on a 3% agarose gel with ethidium bromide staining.

**Table 1:** Amplification conditions, restriction enzymes annealing temperature and fragment size of the studied genes

**Gene**	**Primer sequence**	**SNP**	**AT (°C)**	**Restriction enzyme**	**Fragment sizes (bp)**	**Reference**
*OGG1*	3*'*GTGGATTCTCATCGGTTCG 5*'*	Ser326Cys	58	Fnu4HI	672	Ruyck et al. (2005) [28]
	5*'*CTGTTGCTGTCGAGAATGC 3*'*					
*XRCC1*	3*'*CAAGTACAGCCAGGTCCTAG 5*'*	Arg399Gln	58	BcnI	268	Chiyomaru et al. (2012) [29]
	5*'*CCTTCCCTCATCTGGAGTAC 3*'*					
*XRCC3*	3*'*GCCTGGTGGTCATCGACTC 5*'*	Thr241Met	67	NcoI	552	Krupa et al. (2009) [30]
	5*'*ACAGGGCTCTGGACAGCTCACGTCAC 3*'*					
*XRCC4*	3*'*CTCAGAAGAAATTGTGTATGCT 5*'*	Ile401Thr	52	BseMI	277	Relton et al. (2004) [31]
	5*'*ACCACAAGCAAACTGTGTACAC 3*'*					

*SNP*, single-nucleotide polymorphism; *AT*, annealing temperature in degrees Celsius; *bp*, bases pairs.

### Statistical analysis

The statistical analyses were performed using the Statistical Package SPSS 18.0. A statistically significant value was considered when p ≤ 0.05. Demographic data were presented as mean ± standard deviation. The Mann–Whitney U test was used to compare the means and the chi-square test to compare the proportions of categorical variables in the patients and controls. The distributions of genotypes for each polymorphic site were tested to match the Hardy-Weinberg heredity equilibrium by the chi-square test. The comparison among multiple groups was performed using the nonparametric two-tailed Kruskal-Wallis test with the Dunn correction for multiple comparisons.

The Sample size was calculated for DNA damage by comet assay as the main outcome. Considering a power of 80% and α = 5%, the estimated sample size was fifty subjects [32,33].

## Results

The general characteristics of COPD patients and control group are shown in Table 2. The COPD patients and matched controls were similar in terms of age, gender, ethnicity and BMI, but differed regarding pulmonary function (lower among COPD patients), smoking status, number of cigarettes smoked per year and smoking duration (higher among COPD patients).

**Table 2:** General and clinical characteristic in the COPD patients and control group

	**COPD**	**Control**	***p *value for**
	**n = 51**	**n = 51**	**Mann–Whitney test**
Gender (male)	30	28	>0.05
Ethnicity (white)	45	50	>0.05
Age (years)^a^	65.33 ± 8.91	63.61 ± 9.40	>0.05
BMI (kg/m2)^a^	25.75 ± 5.71	26.82 ± 3.89	>0.05
FEV_1_ (% predicted)	42.90±19.03	86.14±11.72	0.000
FEV_1_/FVC (% predicted)	67.92±19.58	105.24±70.28	0.000
Smoking Status			
Never/ Former/ Current	5/ 34/ 12	22/ 25/ 4	χ^2^ 0.000
Cigarettes-year^b^			
Current smoking	8059 (3650–14600)	7026 (3650–10950)	>0.05
Former smoking	10490 (1095–25550)	5621 (1095–14600)	0.003
Smoking Duration			
>30 years	36	9	χ^2^ 0.000
COPD Status^c^			
Mild	8	-	-
Moderate	16	-	-
Severe	16	-	-
Very Severe	11	-	-

n, sample number; ^a^Data are presented as mean ± SD; ^b^Median (minimum-maximum); ^c^COPD status by GOLD [11] ; *BMI*, body mass index; χ^2^, chi-square test.

The damage index in the alkaline comet assay (COPD 36.71±25.41 *vs* Control 26.65±27.96, *p*=0.005) and in the neutral comet assay (COPD 47.53±32.45 *vs* Control 37.49±38.05, *p *= 0.047) were elevated in COPD patients. In this sense, the percentage of residual DNA damage after 3-h MMS treatment was higher in COPD compared to controls (COPD 145.69±74.11 *vs* Control 54.63±40.32, *p* = 0.000). We stratified our sample in heavy smokers (i.e. ≥ 30 cigarettes per day) and light smokers (i.e.< 30 cigarettes per day) and non-smokers accordance to literature [17,34-38]. In our results no significant difference was found among heavy smokers, only between non-smokers and light smokers (i.e. COPD showed higher DNA damage) for DNA damage index. For BMCyt, no statistically significant difference was measured between case and control group.

No statistical difference was observed between COPD and control group for allelic frequency of 399Gln *XRCC1* (0.343 *vs* 0.314), 326Cys *OGG1* (0.166 *vs* 0.200), 241Met *XRCC3* (0.461 *vs* 0.500), and 401Thr *XRCC4* (0.132 *vs* 0.098) for homozygous genotype. The distribution of allelic frequencies in patients and controls was similar to that found in other population studies in relation to *XRCC1* (Arg399Gln) [6,39,40], *XRCC3* (Thr241Met) [39], and *XRCC4* (Ile401Thr) [41], but lower for *OGG1* (Ser326Cys) [6]. Then, we evaluated the combination of variant genotypes according to the presence of the risk allele. The results are shown in the Table 3. This table shows the effect of risk alleles of the repair genes in BER, HR and NHEJ on the level of different comet assay in COPD and control group.

**Table 3:** Comet assay effects in COPD and controls stratified by genetic polymorphisms in base excision repair (BER), homologous recombination (HR) and nonhomologous DNA end joining (NHEJ) repair genes

	**COPD**	**CONTROL**	p*
**BER**			
Subjects	n= 28	n=26	
***XRCC1-*Arg399Gln**	(ArgGln+GlnGln)	(ArgGln+GlnGln)	
COMET ASSAY			
DI Alkaline	37.29±27.84	21.04±24.45	0.005
DI Neutral	46.50±41.42	29.00±34.46	0.049
DI Residual	150.89±87.99	51.88±44.20	0.000
Subjects	n= 13	n=15	
***OGG1- *Ser326Cys**	(SerCys+CysCys)	(SerCys+CysCys)	
COMET ASSAY			
DI Alkaline	45.54±30.36	41.60±35.05	NS
DI Neutral	60.54±31.63	55.27±47.68	NS
DI Residual	142.38±92.56	56.00±34.98	0.001
**HR and NHEJ**			
Subjects	n= 36	n=36	
***XRCC 3-*Thr241Met**	(ThrMet+MetMet)	(ThrMet+MetMet)	
COMET ASSAY			
DI Alkaline	36.56±24.67	22.50±23.49	0.009
DI Neutral	48.50±30.55	34.53±37.79	0.039
DI Residual	134.75±77.16	54.06±42.88	0.000
Subjects	n= 11	n=07	
***XRCC 4-*Ile401Thr**	(IIeThr+ThrThr)	(IIeThr+ThrThr)	
COMET ASSAY			
DI Alkaline	36.64±23.80	22.36±24.10	NS
DI Neutral	47.36±28.15	36.43±37.30	NS
DI Residual	134.50±80.74	49.02±36.07	0.013

Data are presented as mean ± SD; *DI*, damage index; *NS*, nonsignificant;* Kruskal- Wallis Test.

The DNA damage and residual damage were significantly higher in COPD patients presenting the variant genetic polymorphism in *XRCC1* (Arg399Gln) and *XRCC3* (Thr241Met) than in control group, as viewed by comet assay. The *XRCC4* (Ile401Thr) and *OGG1* (ser326Cys) risk alleles seem to influence only the COPD patient’s residual damage induced by MMS.

No significant difference was observed between groups regarding the frequency of micronuclei and nuclear anomalies by BMCyt assay. So, in order to understand the influence of these genetic polymorphisms in the modulation of DNA damage and progression of COPD, we established a correlation between comet assay and BMCyt assay and pulmonary function in those carrying the risk allele (Table 4).

**Table 4:** Correlations between polymorphisms in DNA repair genes, comet assay and BMCyt assay in COPD patients

**Parameters**	***XRCC1***		***OGG1***		***XRCC3***	
	**Risk allele**		**Risk allele**		**Risk allele**	
	**(ArgGln+GlnGln)**		**(SerCys+CysCys)**		**(ThrMet+MetMet)**	
	Spearman’s rho	*p* value	Spearman’s rho	*p* value	Spearman’s rho	*p* value
% Residual damage- basal DI in alkaline comet assay	−0.497	**0.007**	NS	NS	NS	NS
% Residual damage- basal DI in neutral comet assay	−0.495	**0.007**	NS	NS	NS	NS
% Residual damage- BI	NS	NS	NS	NS	0.350	**0.036**
% Residual damage- KR	NS	NS	NS	NS	0.444	**0.007**
FEV_1_- basal DI in alkaline comet assay	NS	NS	NS	NS	0.336	**0.045**
FEV_1−_ CC	−0.404	**0.029**	NS	NS	NS	NS
FEV_1−_ KR	−0.398	**0.044**	NS	NS	NS	NS
FEV_1_/ FVC- KR	−0.441	**0.024**	NS	NS	−0.408	**0.013**
FEV_1_/ FVC- BUD	NS	NS	NS	NS	−0.357	**0.032**
FEV_1_/ FVC- BI	NS	NS	NS	NS	−0.328	0.051
FVC- CC	−0.428	**0.029**	−0.645	**0.024**	−0.343	**0.041**
FVC- basal DI in alkaline comet assay	NS	NS	NS	NS	0.423	**0.010**
PY- basal DI in neutral comet assay	NS	NS	−0.643	**0.024**	NS	NS
PY- basal DI in alkaline comet assay	NS	NS	−0.616	**0.033**	NS	NS
Basal cell- CC	0.442	**0.032**	NS	NS	NS	NS
Basal cell- MN	NS	NS	NS	NS	0.381	**0.022**
Basal cell- BUD	NS	NS	NS	NS	0.467	**0.004**
Basal cell- KL	NS	NS	−0.666	**0.018**	−0.359	**0.032**

*FEV*_*1*_, forced expiratory volume in one second; *FVC*, forced vital capacity; *DI*, damage index; % Residual damage - calculated for DI after 3 h MMS treatment for each subject taking the value of DI after 1 h MMS treatment as 100%; *BI*, Binucleated cells; *KR*, Karyorrhectic cells; *CC*, Condensed chromatin cells; *BUD*, Nuclear Buds cells; *PY*, Pyknotic cells; *MN*, Micronuclei; *KL*, Karyolytic cells. Statistical analysis was performed by Spearman Correlation test.

**Figure 1:**
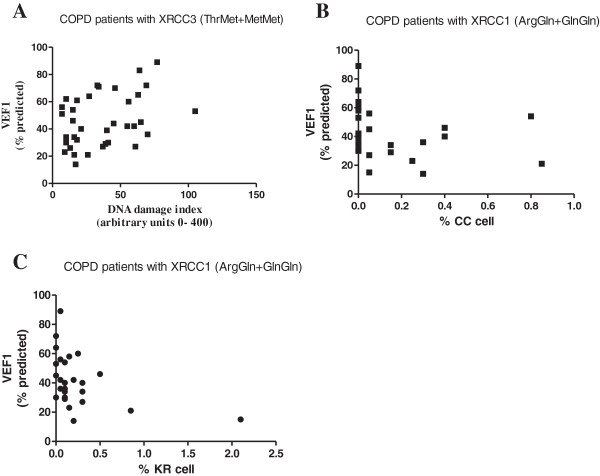
**Correlations between polymorphisms in DNA repair genes, comet assay and BMCyt assay in COPD patients. A)** Damage Index in alkaline Comet assay in blood of COPD patients with variant genotypes in XRCC3 (ThrMet+MetMet). The alkaline basal damage index in COPD patients correlated positively with VEF1 (r 0.336; p=0.045); **B)** % Condensed chromatin cells (CC) in COPD patients with variant genotypes in XRCC1 (ArgGln+GlnGln) correlated negatively with VEF1 (r -0.404; p= 0.029); **C)** % Karyorrhectic cells (KR) in COPD patients XRCC1 (ArgGln+GlnGln) correlated negatively with VEF1 (r -0.398; p= 0.044). COPD patients - black points. Statistical analysis was performed by Spearman’s rho Test.

The alkaline basal damage index in COPD patients correlated positively with CVF and VEF_1_ (Figure 1A). In contrast, negative correlation was found between the basal DNA damage and % residual damage for deficient in *XRCC1* (Arg399Gln) repair. Disease indicators FEV_1_ (Figure 1B and 1C) and FVC correlated negatively with the nuclear anomalies (BUD nuclear, binucleated, condensed chromatin and karyorrhectic cells) for both deficiencies in *XRCC1* (Arg399Gln) and *XRCC3* (Thr241Met) repair.

## Discussion

Our results detected increased basal DNA damage in COPD patients with genetic polymorphisms *XRCC1* (Arg399Gln) and *XRCC3* (Thr241Met) (Table 3). These data are in accordance with previous studies [5,6,10]. The presence of the variant 399Gln in *XRCC1* has been shown to be associated with higher levels of DNA adducts and higher sister chromatid exchange frequencies [6] as well as measurable reduced DNA repair capacity and increased risk of several types of cancers [5]. An association between the irradiation-specific DNA repair rate and the polymorphism in *XRCC1* (Arg399Gln) has been reported in European population [42]. Genetic polymorphism *XRCC3*, which participates in DNA double-strand break repair by homologous recombination pathway, presents a Thr241-Met substitution in exon 7, which was found to be associated with an increase in chromosome deletions using an *in vitro* cytogenetic challenge assay [5]. DNA strand breaks measured by the comet assay are non-specific indicators of transient DNA damage, reflecting equilibrium between damage formation and removal at the particular sampling time [10]. Thus, genetic differences in DNA repair genes, which modify the DNA repair capacity, may directly influence the level of DNA damage [10]. In contrast to our results, Vodicka [43] found no association between comet assay damage index and the *XRCC1* (Arg399Gln) and *XRCC3* (Thr241Met) polymor-phisms probably due *XRCC1* (Arg399Gln) contribute partially to DNA repair capacity and the polymorphisms of other genes could play a role in detecting COPD risk (e.g. *XRCC4* genetic polymorphisms which repairs DNA double-strand breaks by non-homologous end joining [NHEJ] and the completion of recombination events) [11]. Also, *XRCC1* (Arg399Gln) and *OGG1* (Ser326Cys) are suggested to exert combined effect on the development of COPD (i.e. among current/light smokers), and *XRCC1* coordinates and stimulates the OGG1 activity [6]. Results concerning different repair rates between nonsmokers and smokers are inconsistent and the influence of polymorphisms in repair gene has not been established yet [44,45].

DNA effects in the comet assay do not only measure DNA damage but also indicate the DNA repair capacity of the subjects studied. In our study an influence of *XRCC1* (Arg399Gln), *OGG1* (Ser326Cys), *XRCC3* (Thr241Met), and *XRCC4* (Ile401Thr) genetic polymorphisms on DNA repair was found in COPD patients compared to controls, as shown by residual damage (Table 3). These results are in agreement with Hanssen-Bauer [46], which reported that polymorphic variants showed significant and reproducible differences in the pattern of % tail DNA compared to the *XRCC1* Arg/Arg after MMS treatment; initially lower tail. The differences in the repair profiles of the *XRCC1* variants after MMS treatment could be explained by reduced recruitment of these *XRCC1* and interacting proteins to sites of DNA damage, reduced ability to make complexes or interact with DNA glycosylases, and/or reduced efficiency of excision of damaged bases or resolution of strand break intermediates [46]. XRCC1 and XRCC4 proteins are physically bound in mammalian cells with DNA Ligase III and DNA Ligase IV, respectively [47]; *XRCC2* and *XRCC3* genes, members of an emerging family of Rad51- related proteins, have been shown to play an essential role in maintaining chromosome stability in mammalian cells [47]. The *XRCC3* gene is a family member of Rad51-related genes involved in HR and has been associated with a higher incidence of DNA damage [48].

The correlations found in our study showed that the disease progress characterized by higher frequency of cell abnormalities indicatives of cytokinesis defect and/or DNA repair and apoptotic cell death (i.e. Bi, BUD, CC, KR cells) (Table 4). This is the first study to investigate the differences in cellular and nuclear morphology aiming identify potential biomarkers associated with DNA damage, chromosomal instability, cell death, and regenerative potential in COPD patients. Previous studies showed the persistent inflammation in COPD patients leads to higher acetylation of histone resulting in increased transcription of proinflammatory genes, steroid resistance, DNA damage, genomic instability and premature aging [49,50]. Possibly, chromosomal aberrations that directly reflect inappropriate repair of (induced) DNA damage are better suited than comet assay effects for the determination of repair capacities in our study. The higher frequency of condensed chromatin, karyorrhectic, pyknotic and karyolitic cells are indicative of cell death [20]. The apoptosis acts as a surveillance mechanism, eliminating the buccal cells with genetic damage [20]. Similar results were found in studies with subjects exposed to pesticides indicating chromosomal rearrangement [41]. Other study has demonstrated a relationship between genetic susceptibility and biomarkers in occupationally exposed populations [51]. Oxidative stress also damages the DNA repair pathways, such as double-strand break, base excision repair, and nucleotide excision repair, which further cause DNA damage. The cellular senescence and inflammation will form a positive feedback to compromise normal cellular homeostasis [50].

With our results, it is possible to assume an increase in the cancer risk analyzing the correlations that we found between pulmonary function and BMCyt because elevated binucleated cell ratio may be an indicative of higher aneuploidy rate which, in turn, is associated with an increased risk of cancer. The mechanism triggering nuclear bud formation is unknown but may be related to the elimination of amplified DNA or DNA repair complexes [20,52,53]. So, in this time, the BMCyt could be used as a biomarker of monitoring in COPD patients.

However, there are some factors that should be taken into consideration, as the limited database. The sample size may not have been large enough to detect certain differences and there are other genetic polymorphisms that could also influence the repair capacity and consequently the risk for COPD and other repair pathways could also influence repair efficiency.

## Conclusion

Finally, this investigation suggests that basal DNA damage increases, analyzed by Comet and BMCyt assays, in COPD patients with variant genotypes in *XRCC1* (Arg399Gln) and *XRCC3* (Thr241Met). In addition, the induced DNA damage by MMS increases in COPD patients with variant genotypes in *XRCC1* (Arg399Gln), *OGG1* (Ser326Cys), *XRCC3* (Thr241Met) and *XRCC4* (Ile401Thr) showing impairment of DNA repair. Some variant genotypes seem to be related to an increase in Bi, BUD, CC, KR cells, suggesting their possible influence on the background levels of this cytogenetic biomarker and progression of COPD.

## Competing interests

The authors declare that they have no competing interests.

## Authors’ contributions

ALGdS, DJM, ARdMV and JAPH prepared the draft of the manuscript. All the authors collected the data and contributed to the interpretation of results and to the revision of the manuscript. All the authors read and approved the final manuscript.

## Pre-publication history

The pre-publication history for this paper can be accessed here:

http://www.biomedcentral.com/1471-2350/14/93/prepub
